# Short-term fasting lowers glucagon levels under euglycemic and hypoglycemic conditions in healthy humans

**DOI:** 10.1172/jci.insight.169789

**Published:** 2023-06-22

**Authors:** Shana O. Warner, Yufei Dai, Nicole Sheanon, Michael V. Yao, Rebecca L. Cason, Shahriar Arbabi, Shailendra B. Patel, Diana Lindquist, Jason J. Winnick

**Affiliations:** 1 Department of Internal Medicine, Division of Endocrinology, Diabetes and Metabolism, University of Cincinnati College of Medicine, Cincinnati, Ohio, USA.; 2 Department of Pediatrics, Division of Pediatric Endocrinology, Cincinnati Children’s Hospital Medical Center, University of Cincinnati College of Medicine, Cincinnati, Ohio, USA.; 3 Department of Pediatrics, Division of Endocrinology, University of New Mexico, Albuquerque, New Mexico, USA.; 4 Imaging Research Center, Department of Radiology, Cincinnati Children’s Hospital Medical Center, Cincinnati, Ohio, USA.

**Keywords:** Endocrinology, Diabetes

## Abstract

Fasting is associated with increased susceptibility to hypoglycemia in people with type 1 diabetes, thereby making it a significant health risk. To date, the relationship between fasting and insulin-induced hypoglycemia has not been well characterized, so our objective was to determine whether insulin-independent factors, such as counterregulatory hormone responses, are adversely impacted by fasting in healthy control individuals. Counterregulatory responses to insulin-induced hypoglycemia were measured in 12 healthy people during 2 metabolic studies. During one study, participants ate breakfast and lunch, after which they underwent a 2-hour bout of insulin-induced hypoglycemia (FED). During the other study, participants remained fasted prior to hypoglycemia (FAST). As expected, hepatic glycogen concentrations were lower in FAST, and associated with diminished peak glucagon levels and reduced endogenous glucose production (EGP) during hypoglycemia. Accompanying lower EGP in FAST was a reduction in peripheral glucose utilization, and a resultant reduction in the amount of exogenous glucose required to maintain glycemia. These data suggest that whereas a fasting-induced lowering of glucose utilization could potentially delay the onset of insulin-induced hypoglycemia, subsequent reductions in glucagon levels and EGP are likely to encumber recovery from it. As a result of this diminished metabolic flexibility in response to fasting, susceptibility to hypoglycemia could be enhanced in patients with type 1 diabetes under similar conditions.

## Introduction

During a short-term fast (<24 hours), humans are remarkably adept at maintaining their blood glucose level at approximately 90 mg/dL through glycemia-driven minute-to-minute adjustments in islet hormone secretion (1). The 2 islet hormones that figure most prominently in this role are glucagon and insulin, which stimulate and inhibit hepatic glucose production (HGP), respectively, such that it matches the rate of whole-body glucose utilization over time (1). Should a fall in blood glucose occur in healthy humans, it is met with numerous formidable counterregulatory hormone responses (CRRs). First is a decrease in insulin secretion, which can increase HGP such that euglycemia is restored (2–4). Thereafter, glucagon, epinephrine, cortisol, and growth hormone are released in increasing quantities as the glucose level continues to fall (5–12), which has the net effect of stabilizing glycemia by increasing HGP and inhibiting glucose utilization in peripheral tissues (e.g., muscle; ref. 13). In contrast, individuals with type 1 diabetes are unable to regulate their own insulin levels following subcutaneous delivery, and they often exhibit diminished glucagon and epinephrine responses to hypoglycemia (14–20), thereby making them much more vulnerable to low blood glucose. As a result of this heightened susceptibility, fear of debilitating hypoglycemia is a primary limitation to optimal glycemic control in individuals with type 1 diabetes (21), leading to hyperglycemia and greater prevalence of vascular disease and other complications.

Intermittent fasting is a behavior that has been practiced by humans for thousands of years, with modern applications being most closely associated with cultural and religious traditions. More recently, intermittent fasting has become commonplace in secular settings due to the quest for simple dietary strategies that promote and maintain weight loss in the midst of an obesity epidemic (22), and its positive impact on metabolic health and life expectancy (23–29). It has been reported that patients with type 1 diabetes are more vulnerable to hypoglycemia during Ramadan (30), which makes fasting a significant health risk for these individuals. One straightforward way to avoid hypoglycemia during fasting is a change in the timing and/or dose of insulin (31, 32). On the other hand, it has yet to be established whether insulin-independent mechanism(s) related to fasting can also increase susceptibility to hypoglycemia. For example, studies in dogs have shown that acutely increasing liver glycogen content, by an amount similar to what is seen over the course of a day with meals, increased glucagon and epinephrine responses to hypoglycemia, which doubled HGP (13, 33). Likewise, when liver glycogen levels were reduced, the glucagon and HGP responses to hypoglycemia were diminished (33). These findings in animals raise the possibility that fasting-induced reductions in liver glycogen content could also blunt HGP in response to hypoglycemia in humans by lowering the CRR. To this end, we tested the hypothesis that an acute, day-long fast (~22 hours) would impair the hormonal and hepatic responses to insulin-induced hypoglycemia in healthy individuals.

## Results

### Participant characteristics.

Twelve healthy participants (8 F/4 M; mean age: 27 ± 2 years) participated in the study. The average body mass index (BMI) was 22.8 ± 0.7 kg/m^2^ and each participant had a fasting glucose level less than 110 mg/dL at the screening visit (average 89 ± 1 mg/dL; measured with a glucometer; Accu-chek Inform II, Roche Diagnostics).

### Prebreakfast period.

During one study, participants ate breakfast and lunch, after which they underwent a 2-hour bout of insulin-induced hypoglycemia (FED). During the other study, participants remained fasted prior to hypoglycemia (FAST). Prebreakfast levels of glucagon (101 ± 7 and 105 ± 5 pg/mL in FAST and FED, respectively), epinephrine (36 ± 7 and 26 ± 3 pg/mL, respectively), norepinephrine (318 ± 44 and 320 ± 63 pg/mL, respectively), cortisol (33 ± 3 and 31 ± 4 μg/dL, respectively), and nonesterified fatty acids (NEFAs) (150 ± 41 and 158 ± 44 μmol/L) were not different between treatments, whereas growth hormone (GH) was slightly higher in FAST compared with FED (0.49 ± 0.15 and 0.38 ± 0.11 ng/mL, respectively; *P* < 0.05). Plasma glucose, insulin, and liver glycogen content were similar between treatments prior to the breakfast period (Table 1), as were amino acids (Table 2).

### Breakfast period.

As expected, plasma glucose and insulin levels remained unchanged in FAST during the breakfast period (Table 1), although at the cost of a decrease in liver glycogen content over the same period (*P* < 0.05; Table 1). In FED, the breakfast meal contained 761 ± 33 kilocalories, which elevated plasma glucose to a peak at 60 minutes (*P* < 0.05; Table 1). Plasma insulin levels also rose in FED, as expected (*P* < 0.05; Table 1), as did liver glycogen content (*P* = 0.06 compared with FAST; Table 1).

### Lunch period.

Similar to the breakfast period, plasma glucose and insulin remained stable throughout the lunch period in FAST (Table 1) and liver glycogen content was further lowered (*P* < 0.05 compared with baseline value; Table 1). The lunch meal that was consumed during the FED visit contained 769 ± 30 kilocalories. In response to this meal, plasma glucose once again rose in FED, which increased insulin levels over the first hour of the lunch period (Table 1). Prior to the hypoglycemic period, liver glycogen content was 73% higher in FED than it was in FAST (*P* < 0.01 between treatments; Table 1).

### Prehypoglycemia period.

**Figure 1:**
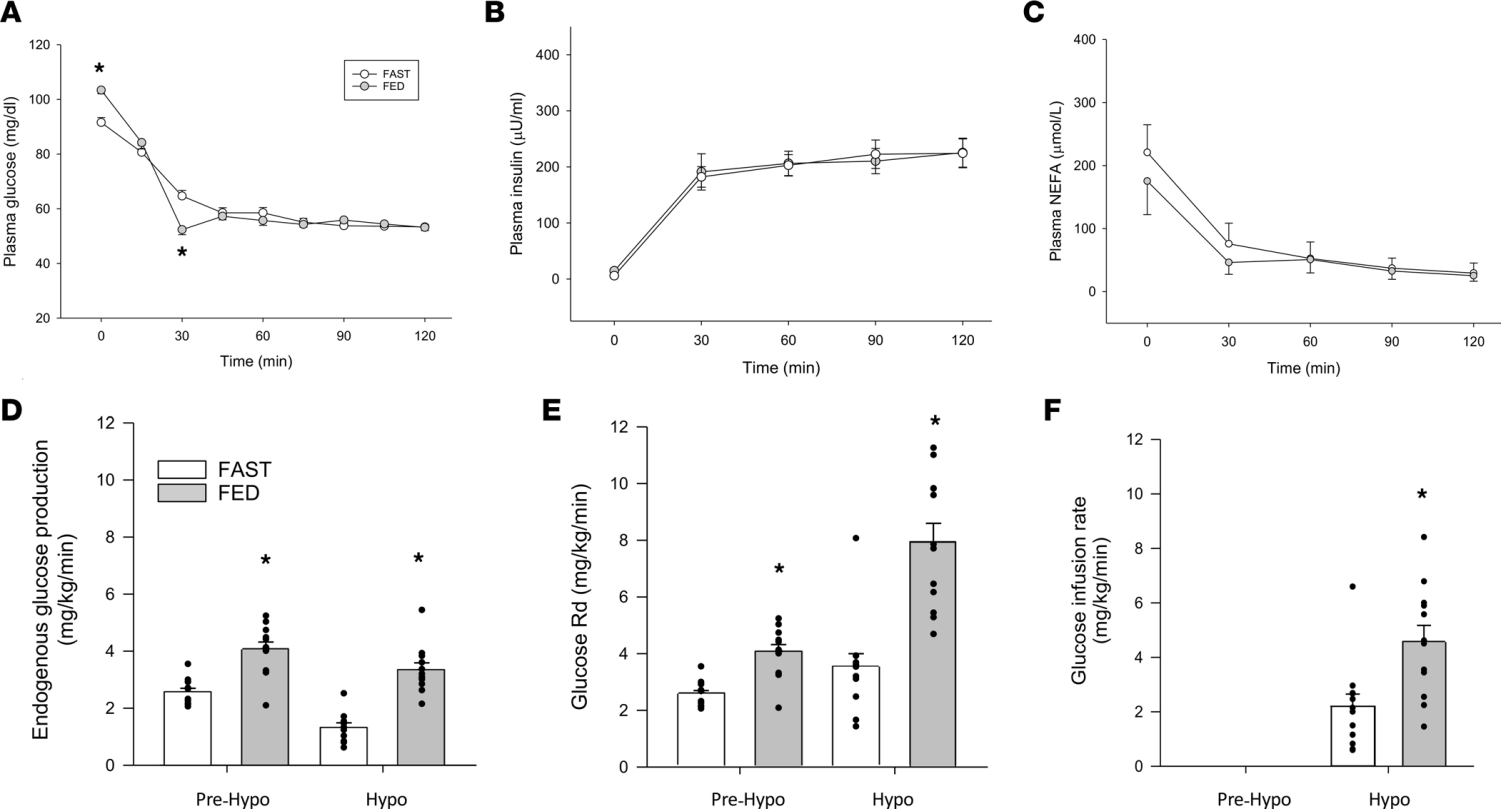
Hormone and substrate responses and glucose kinetics prior to and during insulin-induced hypoglycemia. Plasma glucose (**A**), insulin (**B**), and nonesterified fatty acid (**C**) levels, and endogenous glucose production (**D**), glucose rate of disappearance (**E**), and the exogenous glucose infusion rate (**F**) during the 30 minutes prior to hypoglycemia (Pre-Hypo) and during the final hour of the hyperinsulinemic-hypoglycemic clamp period (Hypo). *n* = 12 (8 F/4 M). *P < 0.05 compared with FAST. Two-way repeated measures ANOVA was used to analyze the data in **A**–**E**, whereas a paired *t* test (2-tailed) was used to analyze the data in **F**.

**Figure 2:**
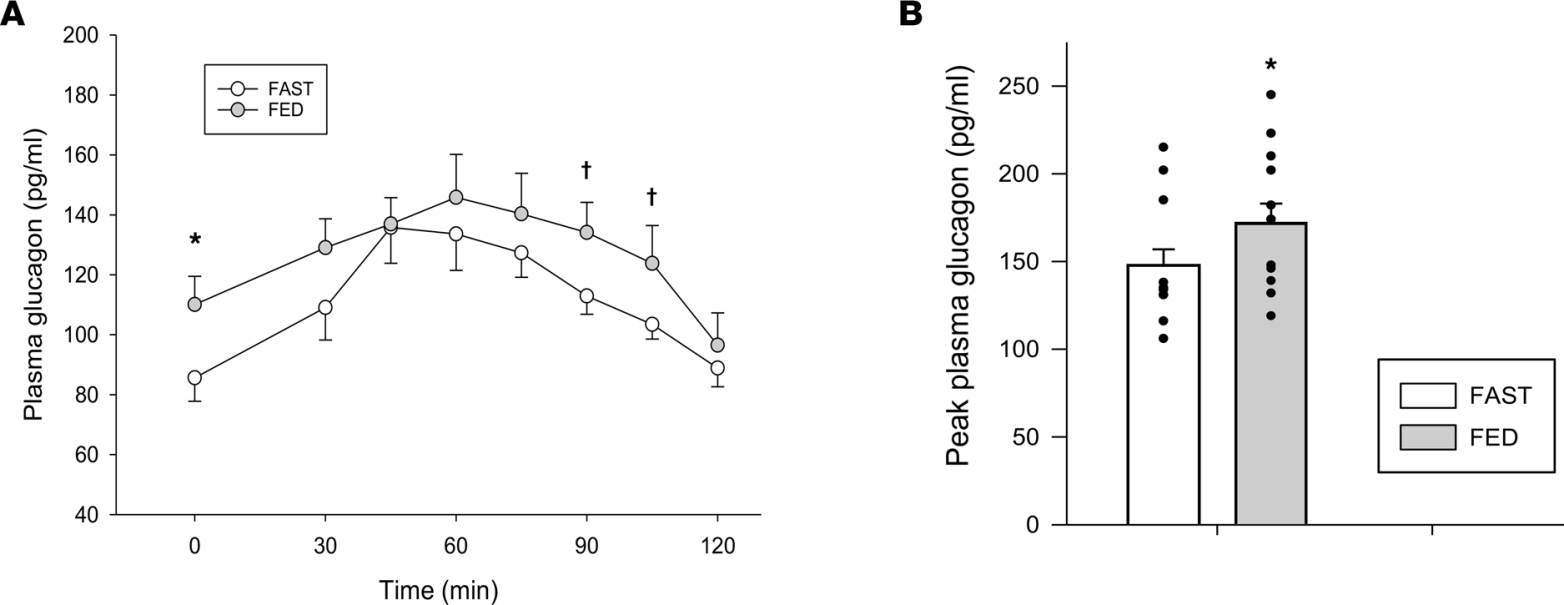
Glucagon levels prior to and during the hyperinsulinemic-hypoglycemic clamp. Plasma glucagon concentrations (**A**) and peak plasma glucagon levels (**B**) during the hyperinsulinemic-hypoglycemic clamp. *n* = 12 (8 F/4 M). *P < 0.05 compared with FAST; ^†^*P* ≤ 0.10 compared with FAST. Two-way repeated measures ANOVA was used to analyze the data in **A**, whereas a paired *t* test (2-tailed) was used to analyze the data in **B**.

Immediately prior to the hypoglycemic period, plasma glucose levels were slightly higher in FED compared with FAST (Figure 1A), whereas the insulin and NEFA levels were similar between treatments (Figure 1, B and C, respectively). The higher glucose level in FED was associated with higher plasma glucagon (Figure 2A), while catecholamine, cortisol, and GH levels were not different between treatments (Table 3). As a result of this hormonal milieu, glucose turnover (i.e., both endogenous glucose production [EGP] and peripheral glucose utilization) was higher in FED compared with FAST (*P* < 0.05 between treatments; Figure 1, D and E). In response to greater EGP, tracer enrichment during the prehypoglycemic period was lower in FED than it was in FAST (*P* < 0.05 between treatments), although the level of enrichment did not change in either treatment during the hypoglycemic period. The plasma levels of most amino acids were not different between treatments, nor was their sum (Table 2); only alanine, methionine, proline, and tyrosine were lower in FAST compared with FED prior to the hypoglycemic period.

### Hypoglycemic experimental period.

As a result of its i.v. infusion, plasma insulin levels were markedly elevated in both treatments (Figure 1B), which lowered NEFA concentrations (Figure 1C). Plasma glucose was slightly lower in FED at the 30-minute time point, after which it became indistinguishable between treatments for the remainder of the hypoglycemic period (Figure 1A). In response to hypoglycemia, cortisol, GH, and catecholamines increased similarly in both treatments over time (*P* < 0.05 for each; Table 3). In contrast, peak glucagon levels (Figure 2B) were lower in FAST and associated with lower EGP during the same period (Figure 1D). Correspondingly, peak glucagon levels were positively correlated with average EGP during the final hour of the hypoglycemic period (*r* = 0.54, *P* < 0.01). Despite similar insulin levels, glucose rate of disappearance (Rd) was attenuated in FAST (Figure 1E), which contributed to a reduction in the need for exogenous glucose to maintain glycemia of approximately 55 mg/dL (Figure 1F). In response to hypoglycemia, no matter the treatment, the plasma level of each amino acid decreased, as did their sum (Table 2). Alanine, proline, and tyrosine remained lower in FAST compared with FED, whereas levels of the other amino acids, their sum, and urea were not different between treatments.

### Between-sex analysis.

Statistical analysis did not reveal any notable between-sex differences during the final hour of the hypoglycemic period for glucagon or glucose metabolism.

## Discussion

Fasting continues to be commonplace for reasons that include religion, weight loss (22, 26), and improved metabolic health (24, 25), but it is also associated with heightened susceptibility to hypoglycemia in patients with type 1 diabetes, making its practice a significant health risk (30). Reducing the basal insulin dose could mitigate this risk, but fasting might also predispose to hypoglycemia by impairing insulin-independent mechanism(s), such as CRRs. In support of this, it has been shown in dogs that acutely lowering liver glycogen content, such as occurs in response to fasting, decreases glucagon and HGP responses to hypoglycemia (33), although it has not yet been determined whether this effect translates to humans. In the current study, we report that the act of fasting for approximately 22 hours, which reduced hepatic glycogen content by 42%, lowered glucagon levels, and diminished EGP in healthy participants under both euglycemic and hypoglycemic conditions. These data support the concept that a reduction in the CRR is an insulin-independent mechanism through which fasting can increase susceptibility to hypoglycemia in humans.

The effect of fasting on glucose metabolism has been studied for many years, yet very little is known about how it impacts the CRR to insulin-induced hypoglycemia. Such studies in rodents can be difficult to translate to human physiology due to their comparatively high rates of whole-body glucose turnover. This markedly increases liver glycogen utilization, thereby hastening depletion of the substrate, which results in lower plasma glucose and enhanced release of counterregulatory hormones, such as glucagon, after an overnight fast (34). In humans, Adamson and colleagues reported that a 72-hour fast lowered glucagon and epinephrine responses to hypoglycemia (35), and also lowered the need for exogenous glucose, which is not incompatible with our data. Importantly, however, such a starvation phenotype also resets whole-body glucose homeostasis by lowering plasma insulin and increasing glucagon and epinephrine levels that, instead of raising plasma glucose, results in a paradoxical decline (36–40). The 22-hour fast our study participants underwent had a negligible impact on plasma glucose, and glucoregulatory hormones remained stable, making it evident that our protocol more closely mimics current applications of fasting by avoiding such a starvation phenotype.

Given that prolonged fasting (>72 hours) increases basal glucagon levels in humans (35, 36), our finding that glucagon was lower in FAST compared with FED prior to the hypoglycemic period is of particular interest. We previously showed that lower hepatic glycogen content was associated with diminished glucagon and HGP responses to insulin-induced hypoglycemia in dogs (33), but the impact of liver glycogen content on islet hormone levels and HGP was not evaluated under euglycemic conditions. Instead, the endogenous secretion of insulin and glucagon was inhibited with somatostatin immediately prior to hypoglycemia and both hormones were matched between groups using intraportal infusions. The current data further this finding by revealing a relationship in humans between fasting, which lowered liver glycogen content by 42%, and decreased glucagon levels and EGP under the unperturbed euglycemic conditions just prior to the hypoglycemic period (i.e., the pancreatic clamp technique was not utilized).

During insulin-induced hypoglycemia, the difference in glucagon between FAST and FED was the same as seen under euglycemic conditions — meaning that the glucagon response to the hypoglycemic challenge did not differ between treatments (Figure 2A). Glucagon secretion in people with type 1 diabetes is present in the basal state, and responsive to the i.v. infusion of secretagogues (e.g., arginine) and amino acid ingestion (14, 41–44). However, these responses can be blunted (44), and glucagon secretion during insulin-induced hypoglycemia is usually impaired, if not completely absent, thereby raising the question as to whether the α cell is an accessible target to reduce hypoglycemic risk. The observation that feeding increased glucagon during the basal, prehypoglycemic period, and that this difference was carried over to and sustained during insulin-induced hypoglycemia, suggests that fasting/feeding is a novel method whereby glucagon secretion can be elicited independently of the hypoglycemia-mediated response. A second important point is patient characteristics; 2 factors that are associated with improved glucagon responses to insulin-induced hypoglycemia in people with type 1 diabetes are older age of diagnosis and shorter duration of the disease, as both are associated with residual β cell function (also referred to as being C-peptide positive; ref. 45). People with type 1 diabetes who are C-peptide positive experience less hypoglycemia than those who are negative for the protein and maintain robust glucagon responses to insulin-induced hypoglycemia (46, 47). If intact glucagon responses to hypoglycemia are required, then it is possible that any positive effect of feeding on CRR would be germane to individuals who are C-peptide positive. In either case, correcting the impaired diurnal pattern of liver glycogen accretion and utilization in people with type 1 diabetes could be of therapeutic value by increasing glucagon levels under nonhypoglycemic conditions that, based on the current data, would persist as the plasma glucose levels fall, and aid in recovery by enhancing HGP. Hepatoselective glucokinase activators, which enhance activity of the rate-limiting enzyme for hepatic glucose uptake and glycogen synthesis, is one class of drug being developed that could potentially achieve this. At this time, one such study has been reported, indicating that chronic liver-specific glucokinase activation can reduce hypoglycemia by 40% in patients with type 1 diabetes, although liver glycogen was not reported (48).

Despite similar insulin levels during the hypoglycemic period, glucose turnover was lower with the FAST treatment. Fasting is known to increase hepatic insulin sensitivity (37–40, 49), although this would not be expected to suppress HGP during hypoglycemia. Rivera and colleagues (50) demonstrated in dogs that hypoglycemia and hyperglucagonemia markedly inhibit insulin action in the liver, making it only a fraction of that seen under euglycemic or hyperglycemic conditions. Consistent with this, dose-response studies have shown that higher insulin levels during hypoglycemia do not further reduce EGP (51–54), suggesting that a modest gain in hepatic insulin sensitivity in FAST would not be expected to account for a 60% reduction in EGP. Instead, the more likely explanation for lower EGP in FAST during the hypoglycemic period is the lower glucagon level. In contrast with the liver, rates of skeletal muscle glucose uptake during hypoglycemia remain closely tethered to insulinemia (37–40, 49). Therefore, fasting-induced peripheral insulin resistance would be expected to attenuate muscle glucose uptake during hypoglycemia, thereby providing a protective effect against a fall in plasma glucose in the face of decreased HGP (i.e., a reduction in the exogenous glucose infusion rate). Whereas this protective effect was significant under the hyperinsulinemic conditions of the current study (~210 μU/mL), its prominence would decline when insulin levels are lower, such as occurs in between meals or during sleep.

Previous work has demonstrated that the ingestion of a mixed meal prior to insulin-induced hypoglycemia also increases glucagon secretion, most likely due to hyperaminoacidemia (44, 55). For this reason, we implemented a 5-hour postlunch absorption period, which has been shown to be of sufficient duration for gastric emptying (56–59) and the rate of appearance of mixed solid meals (60) to be completed in healthy individuals. However, plasma levels of alanine, proline, and tyrosine were higher in FED prior to and during the hypoglycemic period. Neither proline nor tyrosine stimulate glucagon secretion (61, 62), whereas i.v. infusion of alanine has been shown to stimulate glucagon secretion in humans (63). On the other hand, a trebling of plasma alanine concentrations (from ~300 to ~870 μmol/L) had no effect on plasma glucagon levels in healthy individuals (63), suggesting that the small differences in alanine observed between groups (311 vs. 218 μmol/L before hypoglycemia in FED and FAST, respectively, and 210 and 155 μmol/L during hypoglycemia, respectively; Table 2), would not be expected to enhance glucagon secretion.

There are a few limitations of the current study that should be considered. One is our study of healthy control participants instead of patients with type 1 diabetes. However, this was necessary to first establish that our finding of a relationship between liver glycogen concentrations and glucagon secretion in the dog translates to man, after which it will be important to determine whether this relationship remains intact in patients with type 1 diabetes. A second limitation is that we assessed the impact of a single day of fasting on hypoglycemic counterregulation, whereas it is not uncommon for individuals to engage in fasting for numerous consecutive days. It will be important for future studies to further probe this relationship, but based on the current data, a progressive decline in hepatic glycogen would be expected to further diminish the hormonal and hepatic responses to insulin-induced hypoglycemia. A third limitation is that we did not power our studies to determine whether counterregulatory responses to hypoglycemia, in the fasted and fed states, are impacted by sex. It is well known that hormone and glucose turnover responses to hypoglycemia are lower in women than they are in men, although women are at the same time more capable of mobilizing lipid as a fuel substrate (64, 65). Given the uneven distribution of subjects by sex (8 F/4 M), our preliminary conclusion that there was no difference in glucagon or EGP responses between sexes should be interpreted with caution. Finally, we did not measure postexperimental liver glycogen concentrations, so were not able to assess whether reduced glycogenolysis or gluconeogenesis in FAST was responsible for decreased EGP during hypoglycemia.

In summary, our data demonstrate that when compared with consumption of a normal breakfast and lunch, fasting for approximately 22 hours decreased hepatic glycogen content and lowered glucagon and EGP during insulin-induced hypoglycemia. Whereas fasting-induced peripheral insulin resistance might initially buffer against a decline in plasma glucose by reducing peripheral glucose uptake, accompanying reductions in glucagon and EGP during hypoglycemia would be expected to protract the time to recovery. Given the widespread implementation of fasting, the targeting of pathways through which liver glycogen content can be increased under these conditions (e.g., liver-specific glucokinase activators) could prove to be a viable target to enhance metabolic flexibility during hypoglycemia in vulnerable populations, such as those with type 1 diabetes.

## Methods

### Participants.

Healthy males and nonpregnant females of any race or ethnicity and between the ages of 21 and 40 years were eligible to participate. Additional inclusion criteria included a BMI of less than 28 kg/m^2^ and fasting glucose less than 110 mg/dL. Exclusion criteria included cigarette smoking, taking any medication that impacts glucose metabolism, the presence of vascular disease, anemia (hematocrit < 33%), HIV, hepatitis B or C, and a diagnosis of diabetes (type 1 or 2).

### Screening visit.

Each individual participated in 3 study visits (1 screening visit and 2 metabolic studies), all of which were separated by a minimum of 2 weeks. All visits took place at Cincinnati Children’s Hospital Medical Center’s Clinical Research Center (CRC). For the screening visit, participants arrived in the morning after a 12-hour fast, after which written informed consent was given. This was followed by the assessment of anthropometrics and then a blood draw for the determination of fasting plasma glucose and additional inclusion/exclusion criteria (e.g., pregnancy, hematocrit, HIV, hepatitis, etc.). A general health history and physical examination was performed by a physician to ensure that participation was safe for each individual and that they met all eligibility criteria.

### Metabolic studies.

Each participant completed 2 metabolic studies in random order. These 2 visits differed only in that during one visit, participants consumed breakfast and lunch prior to insulin-induced hypoglycemia (FED; described in detail below), and during the other visit they remained fasted prior to the hypoglycemic period (FAST). On the evening before these visits, each participant arrived at the CRC at approximately 1730 hours for blood work, after which they were provided a standardized meal (50% carbohydrate, 30% fat, and 20% protein) that equaled one-third of their daily caloric requirements according to the Mifflin–St. Jeor equation (66). After consuming this meal, the participants left the CRC and remained fasted (except for water) until their return at approximately 0630 hours the following morning.

### Breakfast and lunch periods.

On day 2 of the metabolic studies, participants arrived at the CRC at approximately 0630 hours, after which a catheter was inserted into an antecubital vein for periodic blood draws and kept warm by a heating pad to assist with arterialization. Next, baseline liver glycogen content was assessed using magnetic resonance spectroscopy (MRS; described in detail below). After returning from the MRS suite, participants underwent an 11-hour metabolic study that was divided into 3 periods: a 4-hour breakfast period (0730 to 1130 hours), a 5-hour lunch period (1130 to 1630 hours), and a 2-hour hyperinsulinemic-hypoglycemic clamp period (1630 to 1830 hours). Following a baseline blood draw, participants consumed either a standardized breakfast between 0 and 30 minutes of the breakfast period (FED treatment), or they remained fasted (FAST treatment). Thirty minutes prior to the lunch period, participants underwent a second MRS scan for liver glycogen followed by the consumption of a standardized lunch by FED or continued fasting in FAST. The breakfast and lunch meals were both 60% carbohydrate, 20% fat, and 20% protein, and each contained one-third of the participant’s daily caloric requirements as estimated using the Mifflin–St. Jeor equation (66). Two hours prior to the start of the hypoglycemic period, a second i.v. catheter was placed into the antecubital vein of the contralateral arm, and a primed, continuous infusion of [6,6-^2^H_2_]glucose (Cambridge Isotope Laboratories) was started (0.6 μmol/kg/min) to measure glucose turnover. Approximately 1 hour prior to the hypoglycemic period, a final MRS scan was performed to assess liver glycogen. During the 30 minutes before the hypoglycemic period, blood samples were taken to assess hormones, substrates, and glucose turnover.

### Hypoglycemic experimental period.

At minute 0 of the hypoglycemic period, a primed, continuous insulin infusion was started and infused for 2 hours as previously described (67), and plasma glucose was allowed to fall to 50–55 mg/dL where it was maintained with a variable infusion of 20% dextrose that was labeled with [6,6-^2^H_2_]glucose (3%, w/v) to maintain tracer enrichment. Hypoglycemia of 60 mg/dL or less over the final hour of this period was successfully achieved in 3 of the first 5 research participants with an insulin dose of 60 mU/m^2^/min during both metabolic studies (plasma insulin concentrations were 104.3 ± 2.7 and 96.8 ± 0.7 μU/mL in FAST and FED, respectively). The average plasma glucose level for the other 2 participants receiving this dose was 77 and 67 mg/dL (with no exogenous glucose delivery). For this reason, the latter 2 participants were excluded from our analyses. Thereafter, each participant received an insulin dose of 120 mU/m^2^/min (*n* = 9) during both metabolic studies eliciting plasma insulin values of 254.0 ± 5.5 and 253.2 ± 4.5 μU/mL in FAST and FED, respectively. Statistical analysis using repeated measures ANOVA revealed that there were no differences in hormonal or metabolic responses to hypoglycemia between the 2 insulin doses, so these data were combined to make the total sample size *n* = 12. Blood samples were taken every 5 minutes to assess plasma glucose, and every 15–30 minutes to assess hormones, substrates, and glucose turnover. At the completion of this 2-hour period, the insulin and tracer infusions were discontinued, and each participant was fed a meal. The 20% dextrose infusion was discontinued after stabilization of blood glucose, after which the participant was discharged from the CRC.

### MRS.

Briefly, spectroscopy was performed using a 3-Tesla whole-body MRI system (Philips Achieva) with a ^1^H/^13^C surface coil (Pulse Teq, Ltd) placed over the lateral aspect of each supine participant. A 3-plane localizer was acquired to determine proper coil placement. Glycogen ^13^C spectra were acquired using a nonlocalized proton decoupled ^13^C pulse-acquire sequence over a 15-minute time period (500 ms repetition time, 1800 averages, 8000 Hz bandwidth, 256 points). Spectra were processed with iNMR (Mestrelab Research). Preprocessing included zerofilling to 2048 points, applying 5 Hz line broadening, and Fourier transformation followed by baseline correction using the default parameters. Absolute glycogen concentrations were calculated from the integrated area of the C1-glycogen peak at 100.1 ppm referenced to a glycogen solution of known concentration, corrected for receiver gain and reception sensitivity.

### Biochemical analyses.

Blood samples were collected and processed as previously described (68). Plasma glucose was analyzed using the glucose oxidase method (GM9 Glucose Analyzer, Analox Instruments). Insulin and glucagon were assayed using commercially available radioimmunoassay kits (Millipore). Cortisol (MP Biomedicals) and GH (Thermo Fisher Scientific) were assayed using commercially available ELISA kits. NEFAs were measured using a fluorometric assay (Sigma-Aldrich). Catecholamines and amino acids were assessed using high-performance liquid chromatography and glucose tracer enrichment was assessed using gas chromatography/mass spectrometry.

### Calculations.

Total glucose rate of appearance (Ra) was calculated for each time point by dividing the glucose tracer infusion rate by the tracer enrichment in glucose and is synonymous with the Rd. EGP was calculated by subtracting the exogenous glucose infusion rate from Ra. Prehypoglycemic EGP and Rd (Figure 1, D and E, respectively) are presented as an average of values, taken in 10-minute intervals during the 30 minutes leading up to the hypoglycemic period. Hypoglycemic EGP and Rd were calculated the same way, except that the time interval between samples was 15 minutes and represents the final 60 minutes of the hypoglycemic period.

### Statistics.

All data are presented as mean ± SEM unless stated otherwise. Statistical analyses were performed using SigmaPlot 14.0 (Systat Software, Inc.). Data were analyzed using repeated measures ANOVA with post hoc comparisons made as appropriate using the Student-Newman-Keuls method. A paired *t* test was used to compare peak glucagon responses and glucose infusion rate during the hypoglycemic period.

### Study approval.

The methods of this study were approved by the University of Cincinnati’s Institutional Review Board. Prior to enrollment, all participants were informed of the risks associated with participation and they provided written consent.

## Author contributions

SOW, NS, YD, MVY, SBP, DL, and JJW conducted the studies. SOW NS, YD, DL, and JJW wrote the manuscript and all authors reviewed and edited the final manuscript. RLC was the study coordinator. SA conducted the hormone assays. JJW acquired funding for the studies.

## Figures and Tables

**Table 1:**
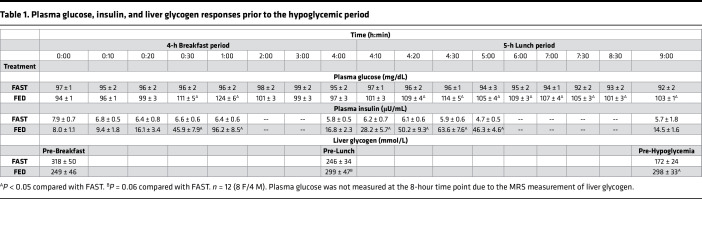
Plasma glucose, insulin, and liver glycogen responses prior to the hypoglycemic period

**Table 2:**
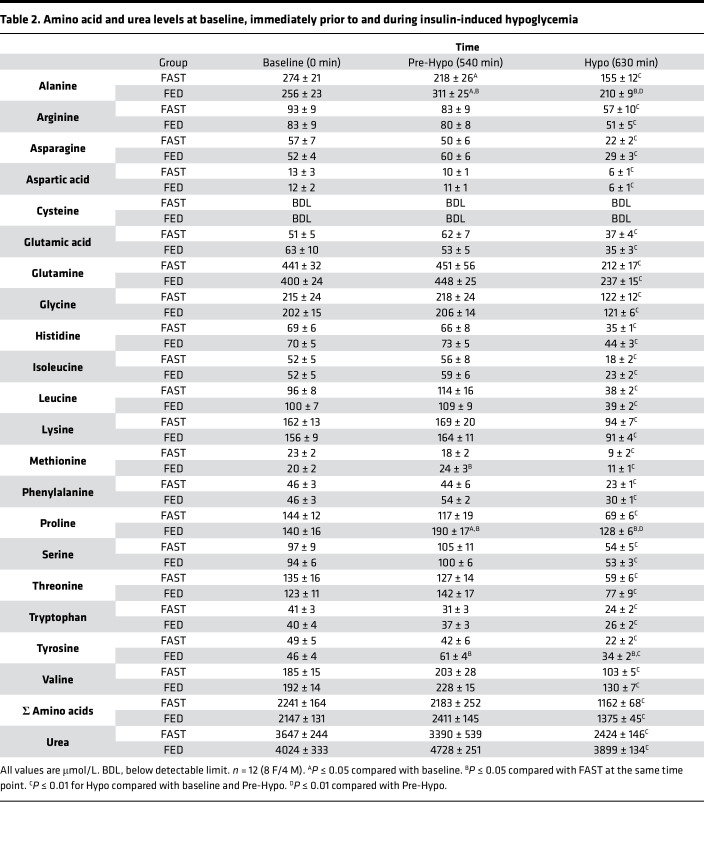
Amino acid and urea levels at baseline, immediately prior to and during insulin-induced hypoglycemia

**Table 3:**
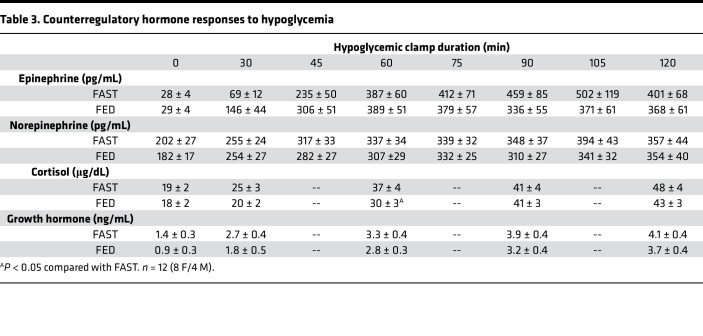
Counterregulatory hormone responses to hypoglycemia

## Version 1 05/11/2023

In-Press Preview

## Version 2 06/22/2023

Electronic publication

## Version 3 06/22/2023

The bar graph in Figure 2 was not completely rendered
